# The prevalence of Tramadol abuse and associated factors among Hawassa University students, Hawassa, Ethiopia

**DOI:** 10.1371/journal.pone.0318634

**Published:** 2025-03-10

**Authors:** Adanech Shifarew Legasse, Wudinesh Tamiru, Fatiya Mohammed, Getu Ataro

**Affiliations:** 1 Department of Anesthesia, Faculty of Medicine, College of medicine and health science, Hawassa University, Hawassa, Sidama, Ethiopia; Ekiti State University College of Medicine, NIGERIA

## Abstract

**Introduction:**

Tramadol abuse is a current public health concern in Ethiopia. Drug abuse including that of tramadol is a significant public health issue that causes health, economic, and social problems to individual drug users, their families, the community, and the entire nation. Therefore this study delivers information regarding current tramadol abuse among students of an Ethiopian University.

**Objective:**

The aim of this study is to assess the prevalence of tramadol abuse and associated factors among Hawassa university students, February to June 2023.

**Methods and materials:**

A cross-sectional study was performed among 402 randomly selected Hawassa university students. Data was collected from participant through self-administered researcher made questionnaire using kobo toolbox software applications. Data from kobo toolbox were directly exported to and analyzed by using SPSS version 26 software. Binary logistic regression was applied to identify the factors associated with tramadol abuse. In the final model, Adjusted Odds Ratio, and 95% CI was used to measure the strength of association and P-value less than 0.05 considered statistically significant.

**Result:**

A total of 402 students were enrolled in this study of which 46 students (11.4%) [95% CI 8.5–14.7] had history of tramadol abuse once in their lifetime with 23.9% of them were active tramadol abuser with some dependence behaviors. The result of multivariate analysis shows that having a friend who use drugs [AOR = 1.691, 95% CI (0.718–3.980), p = 0.041], knowing about tramadol [AOR = 13.766, 95%CI (3.003–63.113), p = 0.001] and having the history of taking tramadol with physicians prescription in their life time [AOR = 7.960, 95%CI (3.603–17.587), P < 0.001] were the significant predictors of tramadol abuse.

**Conclusion:**

The life time prevalence of tramadol abuse among university students is high and it demands attention of government and the general public.

## Introduction

The misuse or abuse of tramadol has emerged as a significant public health issue in Ethiopia, as reported by the Ethiopian Ministry of Health and the Ethiopian Food and Drug Authority (EFDA) [1]. Tramadol was previously available over-the-counter in Ethiopia until November 2022. It is a weak opioid analgesic medication that is prescribed for moderate to severe pain, such as dental pain, labor pain, chronic pain, cancer pain, and acute pain [2,3]. Tramadol’s analgesic effects are attributed to its agonist activity at the Mu opioid receptor, which inhibits serotonin and noradrenaline reuptake and augments central dopamine activation [2]. However, it can cause physical dependence when used consistently for more than a few weeks [4].

Drug abuse is defined as the non-medical use of a substance with the aim of inducing a desired change in mental state or physical performance, such as euphoria in the case of opioids, anesthetics, and sedatives [5]. Tramadol abuse, on the other hand, refers to the use of tramadol without a prescription or using it more frequently, longer, or in higher quantities than recommended [1].

In 2017, approximately 271 million individuals, constituting 5.5% of the global population aged 15 to 64, used drugs, with 35.6 million encountering drug-related problems [6]. Non-medical use of prescription medications poses a significant threat to global public health [3,7]. According to the World Drug Report 2018, tramadol trafficking and non-medical use are the primary drug threats in several African regions [3].

Tramadol usage has significantly increased over time in Egypt and other African countries, necessitating the attention of various authorities [8]. In Egypt, the prevalence of tramadol abuse among university students ranged from 1.9% to 18.9%, high school students at 8.8%, and addiction patients at 49% [ 9,10]. Furthermore, tramadol use ranging from 24.9% to 36.2% was reported among Ghanaian adolescents and drivers [7,11] In Nigeria, the prevalence of tramadol abuse ranged from 3.8% to 54.4% [12].

The socio-environmental characteristics such as conflict in family, peer pressure, having drug abuser friend or family member, having history of smoking or other abuse, higher education, being male and recreational purpose were the commonly reported reasons for tramadol abuse [9,12–14].

Drug abuse, including tramadol use, is a significant public health issue that leads to health, economic, and social problems for individuals, their families, communities, and the nation as a whole. Drug misuse contributes to social issues such as drunk driving, aggression, stress, and child abuse, as well as homelessness, criminality, and missed work [15]. It may also increase the risk of unemployment, physical health issues, dysfunctional social interactions, suicidal tendencies, mental illness, and shorter life expectancy [16]. Studies have also revealed that tramadol use is frequently accompanied by severe side effects, including vomiting, appetite loss, seizures, irritability, and social isolation [13,17,18].

A report of non-medical use or abuse of tramadol was issued by the Ethiopian Ministry of Health and the EFDA in November 2022. In response to concerns about tramadol abuse among young people, the EFDA declared that tramadol should only be prescribed by health professionals using opioid prescriptions, as it is a basic pain medication. However, the extent and prevalence of tramadol abuse in the country have yet to be properly assessed, and no published studies have been found on this issue.

Therefore, this study is timely and essential to provide information on current tramadol abuse and associated factors among University students in Ethiopia. As university students can be a reliable representation of the adult population in the country, the results of this study can provide valuable insights into the status of tramadol in the country. It is crucial to carefully evaluate drug abuse among the young generation and extend significant attention to protect the future of these young drug users/abusers, their families, and the nation as a whole. The findings of this study will contribute to providing current tramadol use status data for the Ethiopian Ministry of Education, Ethiopian Ministry of Health, and health policymakers. Additionally, this study’s results will offer an essential input for future researchers studying the magnitude of tramadol abuse among university students.

The objectives of this study is to determine the prevalence of tramadol abuse and associated factors among Hawassa university students, 2023.

## Materials and methods

### Study area

The study was conducted in selected campuses of Hawassa University, which is located a 275 kilometers south to Addis Ababa city [19].

### Study subject

Study subject of this study is each and every Individual student of Hawassa University that were agreed to be involved in the study

### Study design and period

The cross-sectional study was performed during time period from February to June 2023

### Study methodology

Sample size was calculated by using single population proportion formula, by taking confidence interval of 95% and margin of error of 0.05 and by taking prevalence 36.2% from study done in Ghana [17]. The sample size was 383; after adding 5% non-response rate the final sample size was 402.

Hawassa University has seven different campuses of which four campuses are located in the city and the rest three are out of the city. Therefore four campuses were selected by researchers’ choice, based on their location two located in the city and two out of the city. After selecting the campuses sample size was allocated using allocation formula for each campus based on the number of students found in each campus.

The ethical approval letter was received from institutional review board of Hawassa University College of medicine and health science before starting the study. Study participants were randomly selected from these selected four campuses of Hawassa University undergraduate, graduates and postgraduate students using simple random sampling.

A data was collected by investigator made self-administered questionnaire using kobo collect. The questionnaire were created after studying the literature and consulting experts. After creating account the prepared questionnaire were generated into kobo toolbox server form. Finally the final form of the data collection tool generated into kobo toolbox were deployed to the devices of the data collectors. Oral informed consent was received from each participants (it is already included in kobo collect on first page). Participants who were willing to participate were proceeded to fill the self-administered questionnaire completed by the participants through smart phone and Tablet based kobo collect app. During data collection, students traversing in the university premises en route to and from classrooms, cafeterias, libraries, sport facilities and recreational areas, as well as to and from dormitories, were randomly selected, asked for their informed consent and subsequently included in the study.

Pretest was done on 10% of the sample size that were excluded from the study. The questionnaires that had clarity problem were all edited and then the validated and reliable questions were included as part of the questionnaire.

### Ethics approval and consent to participate.

The ethical approval with the reference number IRB/189/15 was received from institutional review board of Hawassa University College of medicine and health science. Oral informed consent was also taken from all participants. The component of oral informed consent was written and included in the proposal that presented to institution review board and ethical approval was received to proceed.

### Data Management and Analysis

Data in kobo toolbox were directly exported to SPSS version 26 software packages, checked, cleared, and analyzed by using SPSS. The normality for continuous variables was tested by using Shapiro–Wilk test, since the qualitative data in this study were found to be normal distribution and was described in terms of mean and standard deviation. Binary logistic regression was applied to identify the factors associated with tramadol abuse. Bivariable logistic regression was used to identify the candidate variables in the multivariable logistic regression. Variables with p-value less than 0.25 were inserted in to the multivariable logistic regression. Variable Inflation Factor test was used to check multicollinearity. Before fitted in to the final model, model fitness was checked Hosmer Lemeshow goodness of fit test.

Multivariable logistic regression was applied to identify the factors independently associated with tramadol abuse. In the final model, Adjusted Odds Ratio, and 95% CI was used to measure the strength of association and P-value less than 0.05 considered statistically significant.

#### Operational definitions

##### Tramadol abuse:

is non-medical use of tramadol or using tramadol without doctors’ prescription.

## Result

### Socio-demographic characteristics of study participants

A total of 402 students were enrolled in this study, of these almost two third were males (65.7%). The gender of included participants are dominantly male in all campus. The participants were aged 18 to 35 and the mean age was 22 (22 ± 2.882). The majority of these students were single (90%) and most of the respondents were living in campus dormitory (89.3%). [Table 1]

**Table 1:** Socio-demographic characteristics of the study participants.

Variable	Frequency(%)	Mean (x ± SD)
Age		22 ± 2.882
**Gender**		
Female	138 (34.3)	
Male	264 (65.7)	
**Marital status**		
Married	19 (4.7)	
Single	362 (90.0)	
Breakup	21 (5.2)	
**Discipline/ campus**:		
Main	225 (56%)	
Medicine and health	79 (19.7%)	
Awada campus	56 (13.9%)	
Wondogenet campus	42 (10.9%)	
**Residence**		
In dormitory in campus	359 (89.3)	
outside campus rental house	27 (6.7)	
living with family out of campus	16 (4.0)	
**Level of study**		
Undergraduate	358 (89.1)	
Graduate	25 (6.2)	
Post graduate	19 (4.7)	
**Family residence**		
Urban	291(72.4)	
Rural	111 (27.6)	

### The Prevalence of tramadol use and abuse among the study participants

The result shows that out of 402 students enrolled in this study 46 students (11.4%[95% CI 8.5–14.7]of participants) used tramadol without doctor’s prescription once in their lifetime; whereas 16.2% of the participant used tramadol with doctors’ prescription. [Table 2] More than one third (42.3%) of the participants were aware of tramadol. The majority of those who reported tramadol abuse used the tablet preparation form of tramadol (78.26%) whereas the rest of them reported using IV form of tramadol (21.73%) [Table 2].

**Table 2:** The characteristics related with prevalence of tramadol use and abuse among the study participants.

Variables		Frequency (Percentage)
**Do you know tramadol?**	Yes	170 (42.3)
	No	232 (57.7)
**Have you a friend who use drugs?**	Yes	91 (22.6)
	No	311 (77.4)
**Have you a family member who use drug?**	Yes	56 (13.9)
	No	346 (86.1)
Have you ever used substances like Khat, Drug, Alcohol,Cigarette, Hashish,shisha or other in your life?	Yes	71 (17.7%)
	No	331(82.3%)
During your life, have you ever taken tramadol with doctor’s prescription?	Yes	65 (16.2)
	No	337 (83.8)
If you ever take tramadol with doctors’ prescription for how long?	For one day	19 (29.23)
	For <=15 days	21 (32.30)
	For > 15 days	6 (9.23)
	I dont remember	19 (29.23)
During your life, have you ever used any form of tramadol without doctor’s prescription?	Yes	46 (11.4)
	No	356 (88.6)
How much tramadol you use per day? (dose)(for active users)	<100mg	3 (27.27)
	100–300mg	3 (27.27)
	>300mg	5(45.45)
Route of medication	Oral/Tablet form	36 (78.26%)
	Injection	10 (21.76%)
**Substances you take in the last 30days?**	Alcohol beverages	48 (11.9%)
	Tobacco products	4(1.0%)
	Khat	6(1.5%)
	Hashish	1 (0.2%)
	Poly substance	13 (3.2%)
	Tramadol	20 (5.0%)
	Cocaine	6(1.5%)
	Benzodiazepine	2(0.5%)
	Pethidine	1(0.2%)

**Fig 1:**
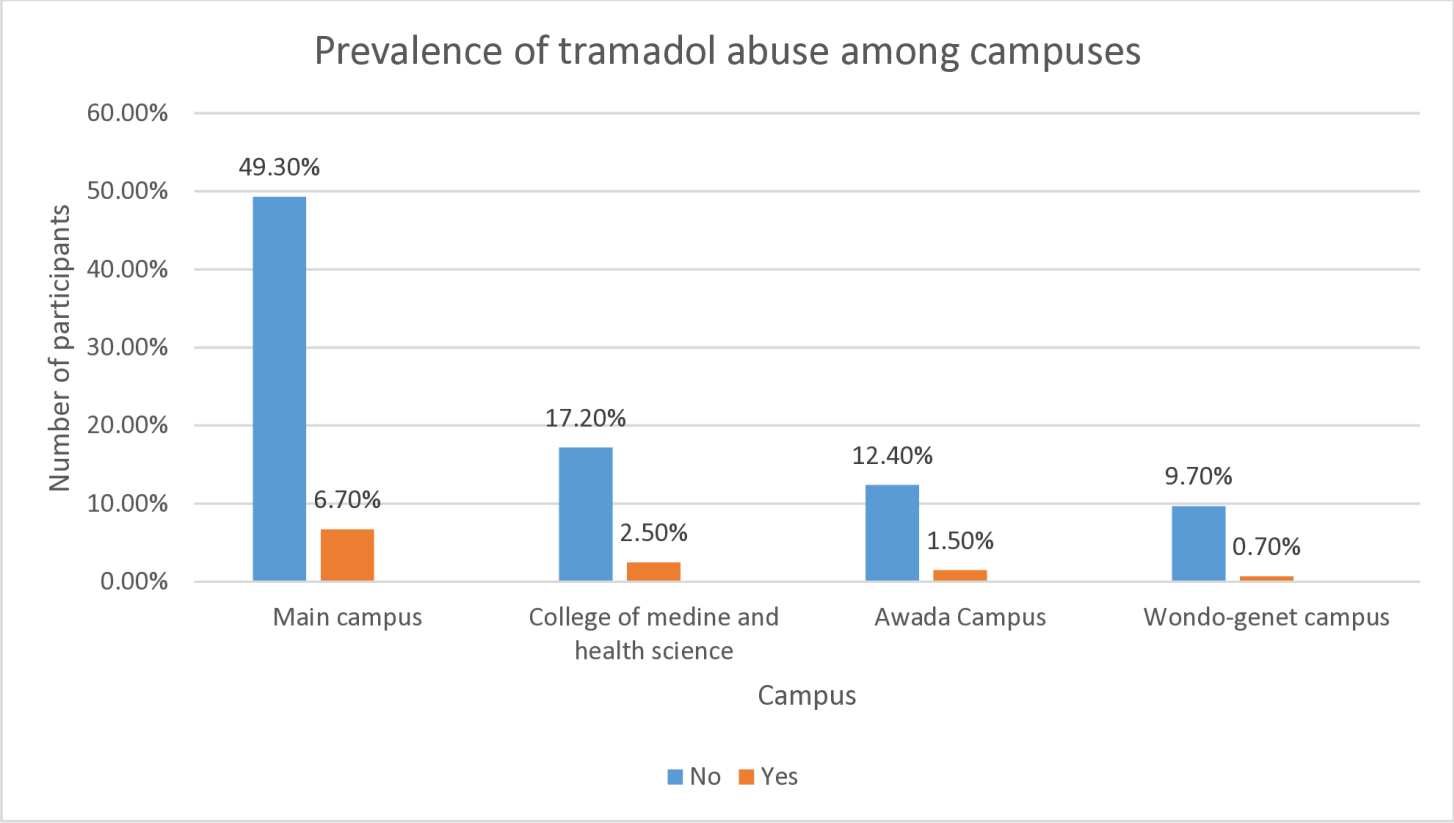
The Prevalence of tramadol abuse distribution among included campuses.

When we see the distribution of the prevalence of tramadol abuse among the included campuses; Main campus took the lead, followed by college of medicine and health science. Among participants from main campus 6.7% reported tramadol abuse [Fig.1].

### Reasons for tramadol abuse among participants who reported tramadol abuse

The major reason that the participants who abuse tramadol reported they used tramadol without physician prescription for the first time was for pain relief (69.9%), followed by peer pressure which accounts for 10.9% of tramadol users. The behavioral reasons that active tramadol abusers repost were strong desire (sense of compulsion) to take tramadol/opioid (63.6%) followed by difficulties in controlling opioid use behavior (18.2%) and physiologic withdrawal state when drug has reduce (18.2%) among the active users. From those who reported tramadol use without doctors’ prescription 23.9% of them were currently active tramadol abuser with some dependence behaviors [Table 3].

**Table 3:** Reasons for tramadol abuse among participants who report history of tramadol use without doctor’s prescription.

Variables	Frequency (%)
**For what reason did you started using tramadol**	
Pain relief	32 (69.6%)
peer pressure	7(15.2%)
Joy seeking or recreational purpose	3 (6.5%)
difficulty of sleeping	3 (6.5%)
low self confidence	1 (2.2%)
**Are you currently use tramadol as a habit actively?**	
Yes	11 (23.9%)
No	35 (76.1%)
**Behavioral reasons for keep using tramadol**	
strong desire(sense of compulsion) to take tramadol	7 (63.6%)
Difficulties in controlling opioid use behavior	2 (18.2%)
physiologic withdrawal state when drug has reduced	2 (18.2%)

### Factors associated with tramadol abuse

The result of multivariate analysis on binary logistic regression shows that having a friend who use drugs (p = 0.041, AOR = 1.691), knowing about tramadol (p = 0.001, AOR = 13.766) and having the history of taking tramadol with physicians prescription in their life time (P < 0.001, AOR = 12.951) were the significantly associated with tramadol abuse [Table 4]. The other factor like gender, marital status, level of study, residential status, discipline, using other psychoactive substance and satisfactions, having a family member who use drugs and satisfaction with university, city and department were not significantly associated with tramadol abuse.

**Table 4:** Factors associated with tramadol abuse among students of Hawassa University 2023.

Variable	Tramadol abuse history		COR [95% CI]	P value	AOR [95%CI]	P value
	Yes (%)	No (%)				
Know about tramadol Yes	44 (10.9%)	135 (33.6%)	40.159 [9.576168.417]	**<0.0001**	13.766 [3.003–63.113]	**0.001**
No	2 (0.5%)	230 (57.2%)	Ref			
Having a friend who use drugs Yes	16 (4%)	75 (28.7%)	0.5 [0.259–0.966]	**0.039**	1.691 [0.718–3.980]	**0.041**
No	30 (7.5%)	281 (69.9%)	Ref			
History of taking tramadol withprescription Yes	32 (8%)	33 (8.2%)		**<0.0001**	7.960 [3.603–17.587]	**<0.0001**
No	14 (3.5%)	323 (80.3%)	22.372[10.857–46.10]			

COR-Crude odds ratio, CI-confidence interval, Ref-reference; AOR- Adjusted odds ratio.

## Discussion

The result of this study shows that from the recruited 402 study participants 11.4% [95% CI 8.5–14.7] of the study participants had history of the tramadol abuse at least once in their life time, of whom 23.9% were active users. Whereas 16.2% of the respondents used tramadol under physician prescription.

The prevalence of tramadol abuse found in present study is in line with findings of study by Bashirian et al. who reported 12.5% prevalence of tramadol abuse among college students in Iran [20]. The finding by Bassiony et al. 2015 in Egypt is also in line with our finding that they reported the prevalence of tramadol abuse was 8.8% among school students [9].

Whereas, The prevalence of tramadol abuse in current study is higher than the prevalence of 1.8% and 1.9% reported on the studies done in Egypt among Sohag University and Zagazig University students respectively [21,22]. It is also higher than the prevalence of history of tramadol abuse in school children reported by Pourmohammadi et al. 2019 in Iran which was 4.71% [23]. Khafagy et al. 2021 found 18.9% prevalence of life time tramadol abuse among Mansoura University students in Egypt [24].which is very higher than current study. The likely explanation for those difference on prevalence of tramadol abuse among these studies might be the variability regarding access to the drug among these countries, since tramadol is not yet under international control.

Study by Lord et al. in 2008 reported the life time opioids abuse among pharmacy students were 8% which was lower than the finding of this present study [25]. The possible explanation regarding the reason for such lower prevalence among pharmacy students may be the contribution of the pharmacological knowledge gained from their study as protective shield against drug abuse. Since our study sample incorporate students from four different campus dispersed among different disciplines and departments.

In our findings 42.3% of the participants know about tramadol abuse. However compared to the above study by Lord et al. 2018 what they know about tramadol might not be with detailed risks and benefits based pharmacological scientific background. This finding agrees with finding by Abdulfetah et al. 2019 done in Egypt who reported more than one third of university students had good knowledge toward tramadol abuse [12].

On the other hand tramadol abuse is significantly associated with knowledge about tramadol use in this present study. Those who had knowledge about tramadol use had almost thirteen times risk of developing tramadol abuse compared to those who had no information about tramadol (P-0.001, OR = 13.766 [3.003–63.113]. This might be due to half of the respondents’ with history of tramadol abuse were from college of medicine and health science and hence majority of health science students know about tramadol use. Khafagy et al. 2021 also reported tramadol as the most commonly used drug among medical students with prevalence of 1.8% [24].

The prevalence of tramadol abuse is significantly associated with having a history of tramadol intake under physician prescription drug (P < 0.0001, AOR = 7.960 [3.603–17.587]). The possible explanation according to WHO studies report is tramadol may cause physical dependence if it is used daily for couple of weeks [3].

Having a friend who practice non-medical drug use is one of the significant associated factors of tramadol abuse in this current study. This result agrees with the finding of the study reported by pourmohammadi et al. 2019 (25). Danso et al. 2021 also reported having a friend who abused drugs significantly associated with tramadol abuse which agrees with the finding of our current study [11]. Peer pressure plays major role in every area of people’s life including that of drug abuse.

Normally tramadol is opioid analgesics that used to treat acute and chronic pain. However there are a misconception as it is perceived by recreational users as a means of improving mood and boosting energy [3]. In this study the reasons that the participants with history of tramadol abuse started using tramadol was majorly for pain relief constituting the 69.6% of the abusers, but also peer pressure (15.2%), joy seeking or recreational purpose (6.5%), difficulty of sleeping (6.5%) and low self-confidence(2.2%) were the reasons some of them started using tramadol.

The predominant reasons active tramadol abuser were currently keep taking this drug was the strong desire (sense of compulsive urge or craving) to take tramadol followed by recreational purpose. This is consistent with finding by Ibrahim et al. 2017 in Nigeria that reported compulsive urge or craving for the drug as third reason to continual abuse of tramadol [12]. Another Study done in Egypt by Mohamed et al. 2022 reported that the most common reason for tramadol abuse was for the pleasure effect to have good time which the similar with the second common reason in this current study [9].

Almost 36 (9.0%) participants or more than two third of the participants with history of tramadol abuse used the oral or tablet form of tramadol. The study by Ibrahim et al. 2017 reported 96.1% of participants used tablet form of tramadol which is higher than our finding [12]. The likely reasons for this difference may be tramadol (all formulation) was over-the-counter medicine in Ethiopia till the time of announcement by EFDA in November 2022 [1].

This study is not without limitations. This study is done only in one University it is better if it incorporate different universities in Ethiopia just to generalize the prevalence of tramadol among Ethiopian university students at national level. The other limitations of this study is its cross-sectional nature. Even though this study has so many limitations it has its own qualities and strengths. From these good qualities the data collection process was done by using software application called Kobo Tool box, which is simple, easy and amazing technology that reassure the participants to give the right information without any fear of privacy.

### Conclusion

The life time prevalence of tramadol abuse among university students is high and it demands attention of government and general public. The outcome of this study shows that the prevalence of life time tramadol abuse was 11.4%, of whom 23.9% of them were active users. Tramadol abuse was significantly associated having a friend who use drugs, knowing about tramadol and having the history of taking tramadol under physician’s prescription in their life time.

### Recommendation

We recommend the future researcher to do large nationwide multicenter study. We recommend the higher education administrates to give health education on tramadol abuse risks and consequences for university students. We also recommend health professional to be cautious and responsible regarding rational prescription of tramadol.

## Supporting Information

S1 Data Table 1.1. Age in years. Table 1.2. Gender. Table 1.3. Marital status. Table 1.4. Residential status (current). Table 1.5. Place of residence of family (previous). Table 1.6. Monthly income/ allowance source. Table 1.7. Discipline. Table 1.8. Semester. Table 1.9. Level of study. Table 2.1. Have you ever tried substance like khat, drug alcohol, cigarette smoking. Table 2.2. If yes, which of the following substance did you ever used or currently using? Table 2.3. Are you currently had a habit of using any drug yourself without Dr prescription? Table 2.4. If yes, which of the following drug. Table 2.5. Do you know tramadol. Table 2.6. During your life time did you ever take tramadol with Drs prescription? Table 2.7. If yes for how long? Table 2.8. During your life time did you ever take tramadol with out Drs prescription? Table 2.9. If yes what reason? Table Other reason you use tramadol. Table 2.10. Are you currently using tramadol as a habit? Table 2.11. If yes how often do you use tramadol. Table 2.12. For how long did you used tramadol. Table 2.13. How much tramadol you use perday? Table how do you use tramadol or other drug? Table 2.15. For what reason you currently using tramadol? Table 2.16. Have you ever used pethidine? Table 2.17. If you are frequent tramadol or other opioid user, encircle the behavior you had from the following? (Don’t be confused tramadol is opioid). Table 2.18. Do you have a friend who use drug? Table 2.19. Have you Family member who use drugs. Table 3.1 Satisfaction with the city of study. Table 3.2. Satisfaction with University. Table 3.3. Satisfaction with Discipline/department. Table 3.4. Monthly income. (PDF)
