# Supplementary material for: The prevalence of Tramadol abuse and associated factors among Hawassa University students, Hawassa, Ethiopia
Source: PLoS One. 2025 Mar 10;20(3):e0318634. doi: 10.1371/journal.pone.0318634 (PMC11892866; doi:10.1371/journal.pone.0318634)
Supplement: S1 Data — (PDF) [file pone.0318634.s001.pdf]

Clone of Drug data collection A

Custom Reports

Clone of Drug data collection

This is an automated report based on raw data submitted to this project. Please conduct proper data cleaning prior to using the graphs and figures used on this page.

1.1. Age in years

TYPE: INTEGER. 391 out of 391 respondents answered this question. (0 were without data.)

Mean	Median	Mode	Standard deviation
52.23	22.00	22.00	242.70

1.2. Gender

TYPE: SELECT_ONE. 391 out of 391 respondents answered this question. (0 were without data.)

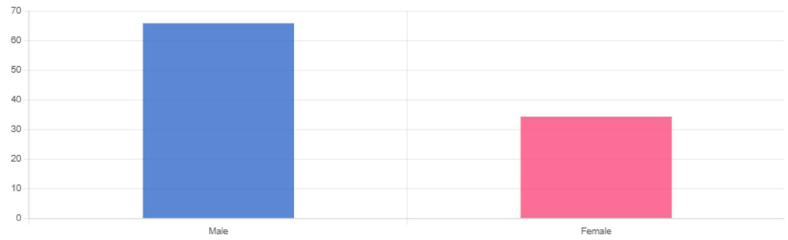

Value	Frequency	Percentage
Male	257	65.73
Female	134	34.27

1.3. Marital status

TYPE: SELECT_ONE. 391 out of 391 respondents answered this question. (0 were without data.)

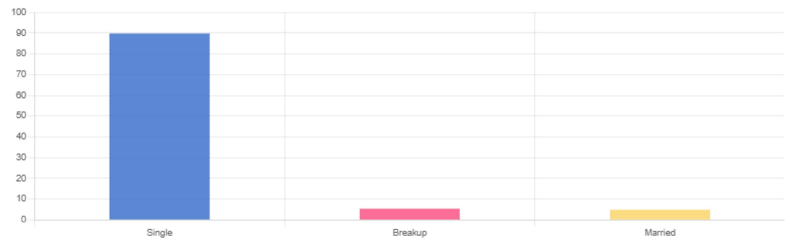

Value	Frequency	Percentage
Single	351	89.77
Breakup	21	5.37
Married	19	4.86

1.4. Residential status

TYPE: SELECT_MULTIPLE. 391 out of 391 respondents answered this question. (0 were without data.)

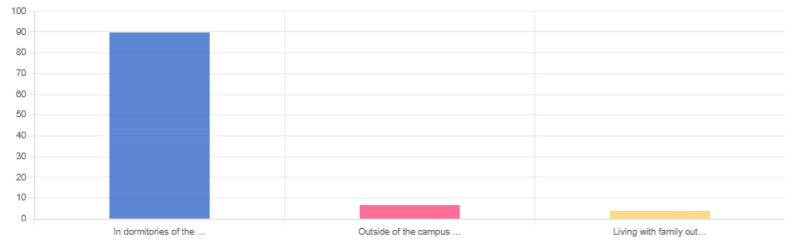

Value	Frequency	Percentage
In dormitories of the campus	351	89.77
Outside of the campus in rental house	26	6.65
Living with family out side campus	15	3.84

1.5. Place of residence of family

TYPE: SELECT_ONE. 391 out of 391 respondents answered this question. (0 were without data.)

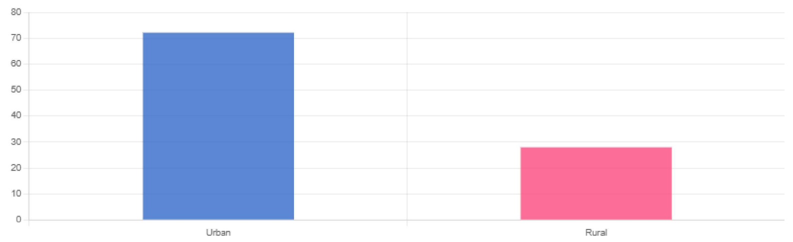

Value	Frequency	Percentage
Urban	282	72.12
Rural	109	27.88

1.6. Monthly income/allowance source

TYPE: SELECT_MULTIPLE. 282 out of 391 respondents answered this question. (109 were without data.)

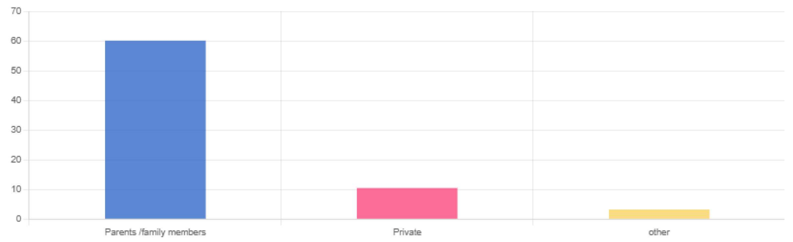

Value	Frequency	Percentage
Parents /family members	235	60.1
Private	41	10.49
other	13	3.32

1.7. Discipline/Campus

TYPE: SELECT_ONE. 391 out of 391 respondents answered this question. (0 were without data.)

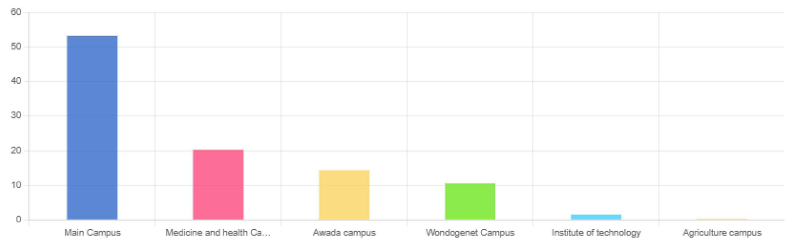

Value	Frequency	Percentage
Main Campus	208	53.2
Medicine and health Campus	79	20.2
Awada campus	56	14.32
Wondogenet Campus	41	10.49
Institute of technology	6	1.53
Agriculture campus	1	0.26

1.8. Semester

TYPE: SELECT_ONE. 391 out of 391 respondents answered this question. (0 were without data.)

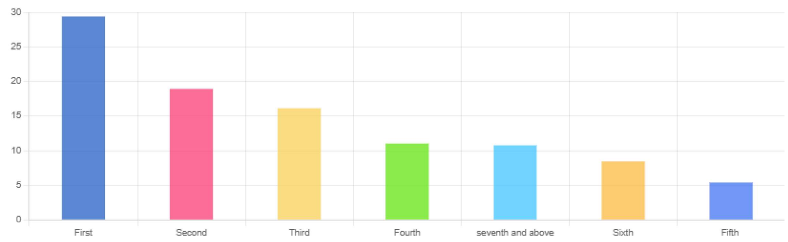

Value	Frequency	Percentage
First	115	29.41
Second	74	18.93
Third	63	16.11
Fourth	43	11
seventh and above	42	10.74
Sixth	33	8.44
Fifth	21	5.37

1.9. level of study

TYPE: SELECT_ONE. 391 out of 391 respondents answered this question. (0 were without data.)

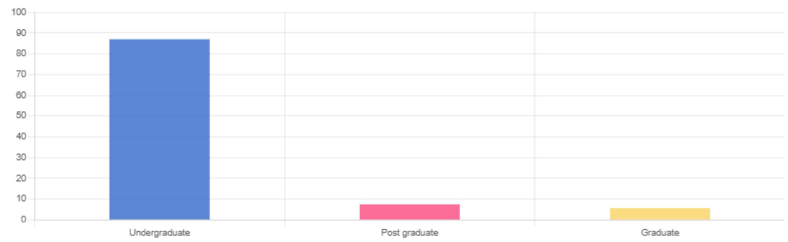

Value	Frequency	Percentage
Undergraduate	340	86.96
Post graduate	29	7.42
Graduate	22	5.63

2.1. Have you ever used substances like Khat, Drug, Alcohol, Cigarette, Hashish,shisha or other in your life?

TYPE: SELECT_ONE. 391 out of 391 respondents answered this question. (0 were without data.)

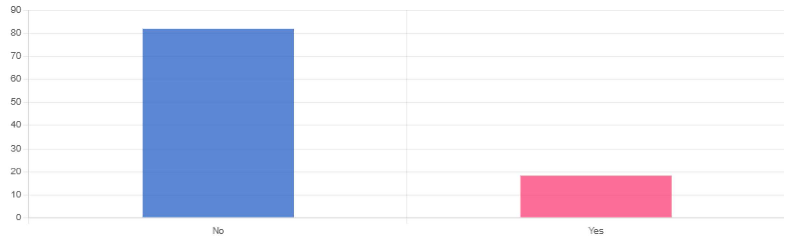

Value	Frequency	Percentage
No	320	81.84
Yes	71	18.16

2.2. If yes, which of the following substance did you ever used (or currently using)?

TYPE: SELECT_MULTIPLE. 71 out of 391 respondents answered this question. (320 were without data.)

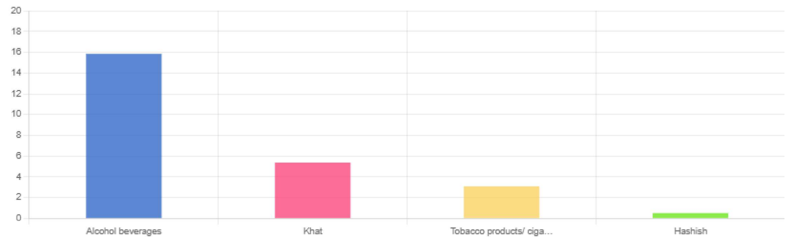

Value	Frequency	Percentage
Alcohol beverages	62	15.86
Khat	21	5.37
Tobacco products/ cigarette	12	3.07
Hashish	2	0.51

2.3. Are you currently had a habit of using any drug by yourself without Dr's Prescription?

TYPE: SELECT_ONE. 391 out of 391 respondents answered this question. (0 were without data.)

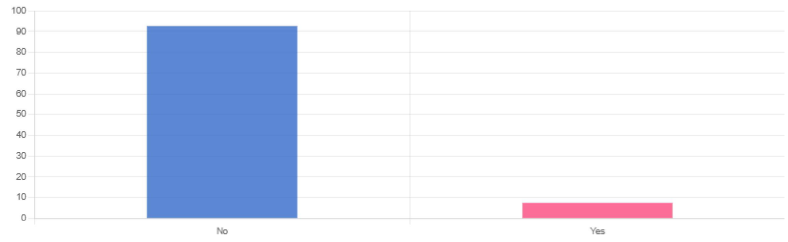

Value	Frequency	Percentage
No	362	92.58
Yes	29	7.42

2.4. If Yes which of the following drugs are you currently use?

TYPE: SELECT_MULTIPLE. 29 out of 391 respondents answered this question. (362 were without data.)

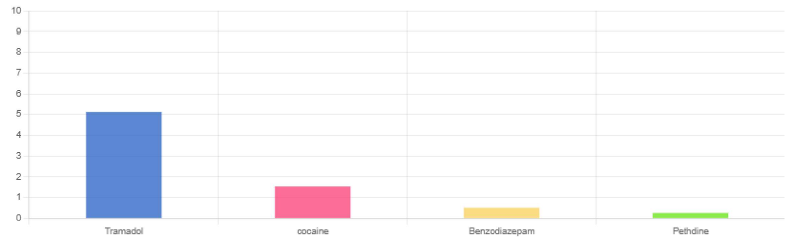

Value	Frequency	Percentage
Tramadol	20	5.12
cocaine	6	1.53
Benzodiazepam	2	0.51
Pethdine	1	0.26

2.5. Do you know tramadol?

TYPE: SELECT_ONE. 391 out of 391 respondents answered this question. (0 were without data.)

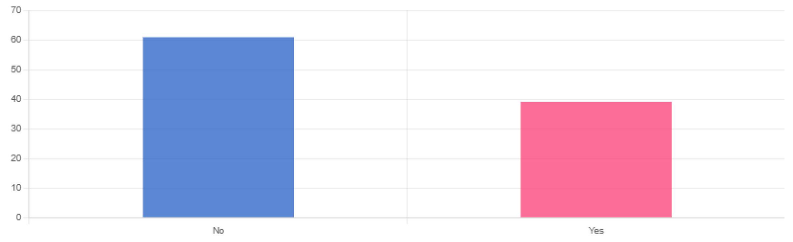

Value	Frequency	Percentage
No	238	60.87
Yes	153	39.13

2.6. During your life, have you ever taken tramadol with doctor’s prescription?

TYPE: SELECT_ONE. 391 out of 391 respondents answered this question. (0 were without data.)

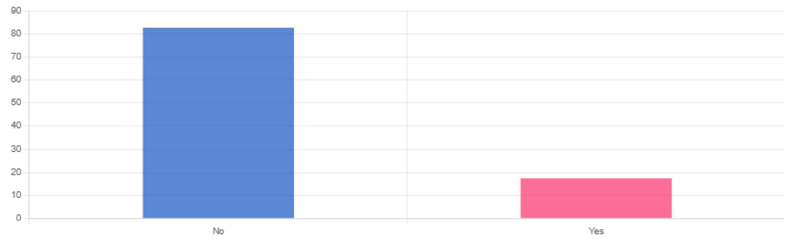

Value	Frequency	Percentage
No	323	82.61
Yes	68	17.39

2.7. If Yes, for how long?

TYPE: SELECT_ONE. 68 out of 391 respondents answered this question. (323 were without data.)

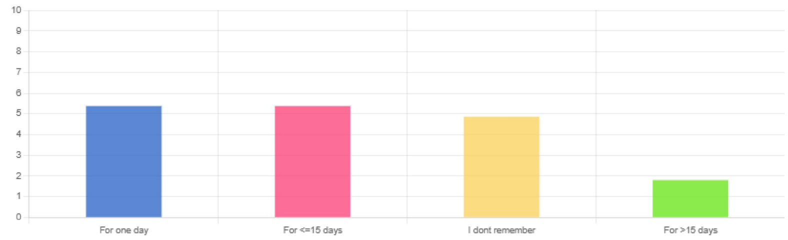

Value	Frequency	Percentage
For one day	21	5.37
For <=15 days	21	5.37
I dont remember	19	4.86
For >15 days	7	1.79

2.8. During your life, have you ever used any form of tramadol without doctor’s prescription?

TYPE: SELECT_ONE. 391 out of 391 respondents answered this question. (0 were without data.)

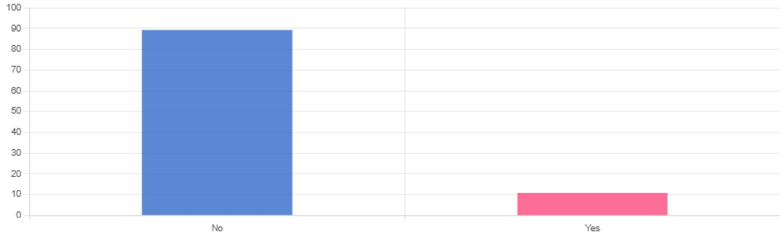

Value	Frequency	Percentage
No	349	89.26
Yes	42	10.74

2.9. If YES, For what reason did you used tramadol? (You can answer more than one)

TYPE: SELECT_MULTIPLE. 42 out of 391 respondents answered this question. (349 were without data.)

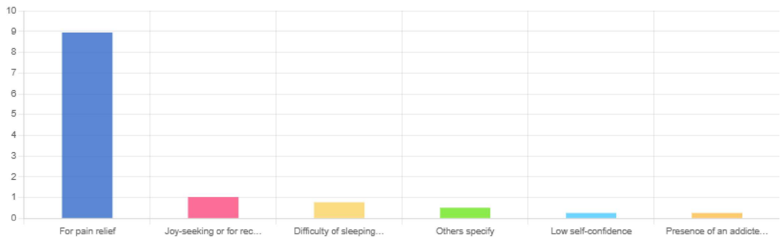

Value	Frequency	Percentage
For pain relief	35	8.95
Joy-seeking or for recreational purpose	4	1.02
Difficulty of sleeping/ sleep related problems	3	0.77
Others specify	2	0.51
Low self-confidence	1	0.26
Presence of an addicted person in the family or friend	1	0.26

Others reason you used tramadol specify

TYPE: TEXT. 2 out of 391 respondents answered this question. (389 were without data.)

Value	Frequency	Percentage
P	1	0.26
headache	1	0.26

2.10. Are you currently use tramadol as a habit ?

TYPE: SELECT_ONE. 391 out of 391 respondents answered this question. (0 were without data.)

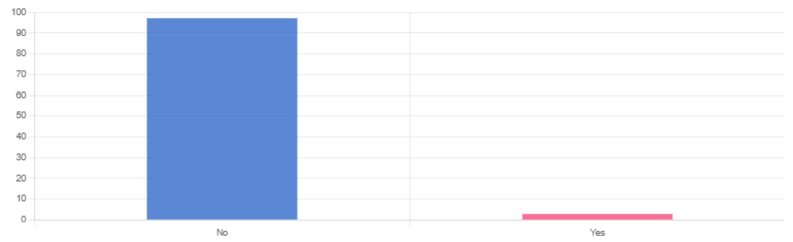

Value	Frequency	Percentage
No	380	97.19
Yes	11	2.81

2.11. if yes, How often do you use tramadol ? (Frequency)

TYPE: SELECT_ONE. 11 out of 391 respondents answered this question. (380 were without data.)

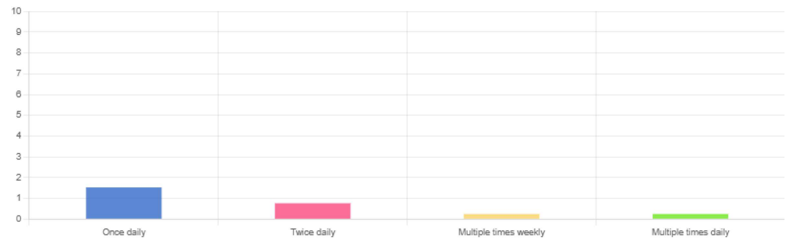

Value	Frequency	Percentage
Once daily	6	1.53
Twice daily	3	0.77
Multiple times weekly	1	0.26
Multiple times daily	1	0.26

2.12. For how long did you use tramadol?(since when you started using)

TYPE: TEXT. 42 out of 391 respondents answered this question. (349 were without data.)

Value	Frequency	Percentage
2	7	1.79
1	6	1.53
12	2	0.51
a Few Times	1	0.26
some Time	1	0.26
1day .1Dose	1	0.26
3x	1	0.26
some Times	1	0.26
When I fell pain	1	0.26
7	1	0.26
No	1	0.26
1 years ago	1	0.26
<15	1	0.26
10	1	0.26
Five years	1	0.26
15 years	1	0.26
Tow thousand ten	1	0.26
Two years	1	0.26
3	1	0.26
one day only	1	0.26
simce i was 5 years old	1	0.26
For one day only	1	0.26
three	1	0.26
Once	1	0.26

4	1	0.26
8	1	0.26
18	1	0.26
since gread 9	1	0.26
twice in a months	1	0.26
when i feel pain	1	0.26

2.13. how much tramadol you use per day?

TYPE: INTEGER. 11 out of 391 respondents answered this question. (380 were without data.)

Mean	Median	Mode	Standard deviation
4.27	2.00	*	5.78

2.14. How do you use tramadol/pethidine or other drug? (Route) or in which form?

TYPE: SELECT_MULTIPLE. 42 out of 391 respondents answered this question. (349 were without data.)

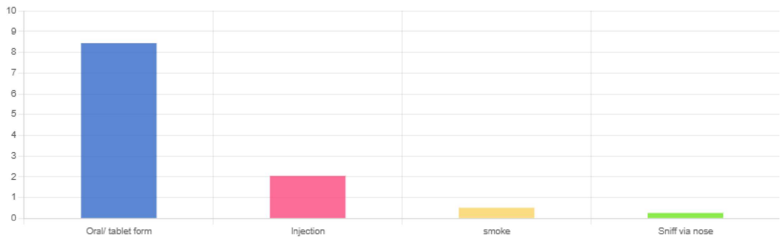

Value	Frequency	Percentage
Oral/ tablet form	33	8.44
Injection	8	2.05
smoke	2	0.51
Sniff via nose	1	0.26

2.15. For what reason you currently use tramadol? (You can select more than one)

TYPE: SELECT_MULTIPLE. 11 out of 391 respondents answered this question. (380 were without data.)

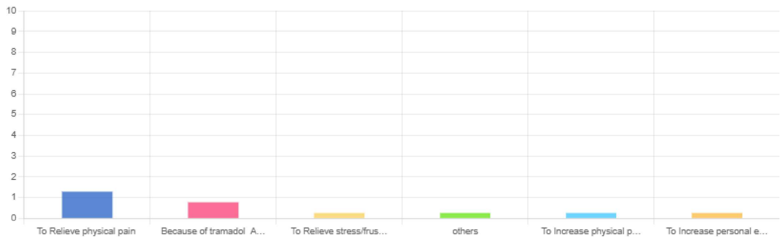

Value	Frequency	Percentage
To Relieve physical pain	5	1.28
Because of tramadol Addiction/ its CRAVING (compulsive urge)	3	0.77
To Relieve stress/frustration	1	0.26
others	1	0.26
To Increase physical performance/ to decrease tiredness (fatigue)	1	0.26
To Increase personal euphoria (happiness)/ for recreational purpose or to improve mood	1	0.26

2.16. Have you ever used pethidine ?

TYPE: SELECT_ONE. 42 out of 391 respondents answered this question. (349 were without data.)

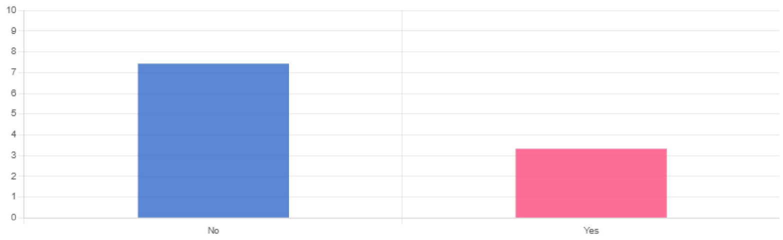

Value	Frequency	Percentage
No	29	7.42
Yes	13	3.32

2.17. . If you are frequent tramadol or other opioid user, encircle the behavior you had from the following? (Don’t be confused tramadol is opioid)

TYPE: SELECT_MULTIPLE. 11 out of 391 respondents answered this question. (380 were without data.)

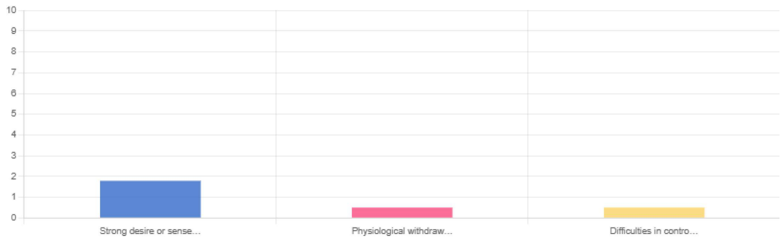

Value	Frequency	Percentage
Strong desire or sense of compulsion to take tramadol/opioids	7	1.79
Physiological withdrawal state when drug use has stopped/reduced	2	0.51
Difficulties in controlling opioid-use behaviors in terms of the onset, termination or levels of use	2	0.51

2.18. Have you a friend who use drugs?

TYPE: SELECT_ONE. 391 out of 391 respondents answered this question. (0 were without data.)

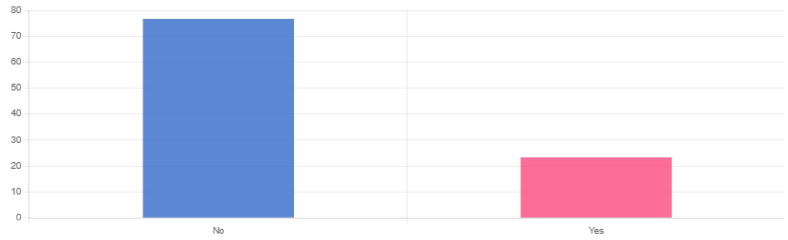

Value	Frequency	Percentage
No	300	76.73
Yes	91	23.27

2.19. Have you a family member who use drug?

TYPE: SELECT_ONE. 391 out of 391 respondents answered this question. (0 were without data.)

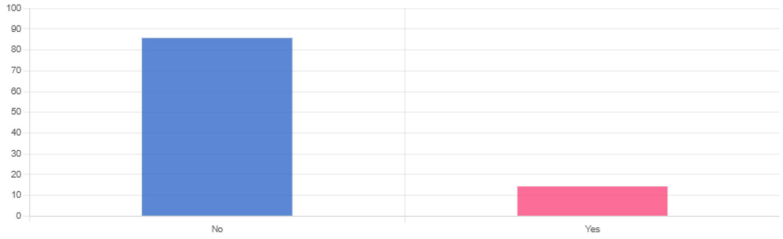

Value	Frequency	Percentage
No	335	85.68
Yes	56	14.32

Satisfaction with city of study

TYPE: SELECT_ONE. 391 out of 391 respondents answered this question. (0 were without data.)

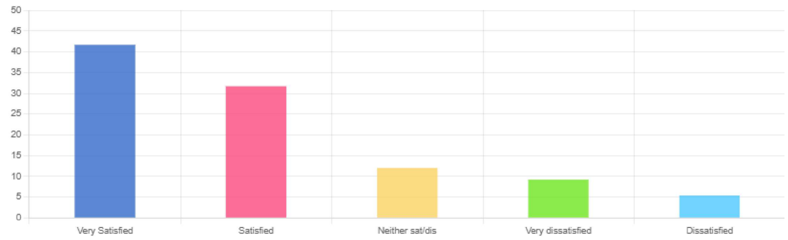

Value	Frequency	Percentage
Very Satisfied	163	41.69
Satisfied	124	31.71
Neither sat/dis	47	12.02
Very dissatisfied	36	9.21
Dissatisfied	21	5.37

Satisfaction with University

TYPE: SELECT_ONE. 391 out of 391 respondents answered this question. (0 were without data.)

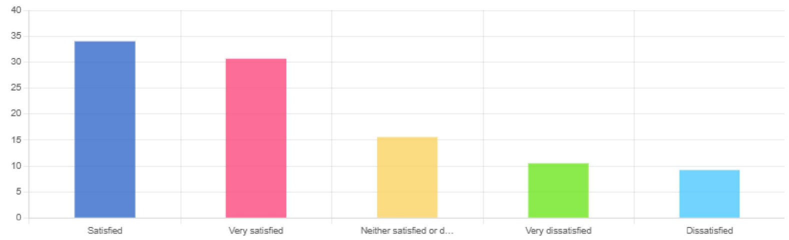

Value	Frequency	Percentage
Satisfied	133	34.02
Very satisfied	120	30.69
Neither satisfied or dissatisfied	61	15.6
Very dissatisfied	41	10.49
Dissatisfied	36	9.21

Satisfaction with department

TYPE: SELECT_ONE. 391 out of 391 respondents answered this question. (0 were without data.)

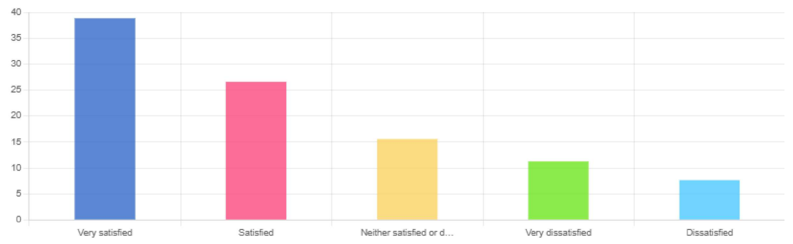

Value	Frequency	Percentage
Very satisfied	152	38.87
Satisfied	104	26.6
Neither satisfied or dissatisfied	61	15.6
Very dissatisfied	44	11.25
Dissatisfied	30	7.67

Income_Monthly_allowance

TYPE: INTEGER. 109 out of 391 respondents answered this question. (282 were without data.)

Mean	Median	Mode	Standard deviation
2482.16	1000.00	1000.00	3257.60
